# Supplementary material for: The phased pan-genome of tetraploid European potato
Source: Nature. 2025 Apr 16;642(8067):389–97. doi: 10.1038/s41586-025-08843-0 (PMC12158759; doi:10.1038/s41586-025-08843-0)

Chromosome 2

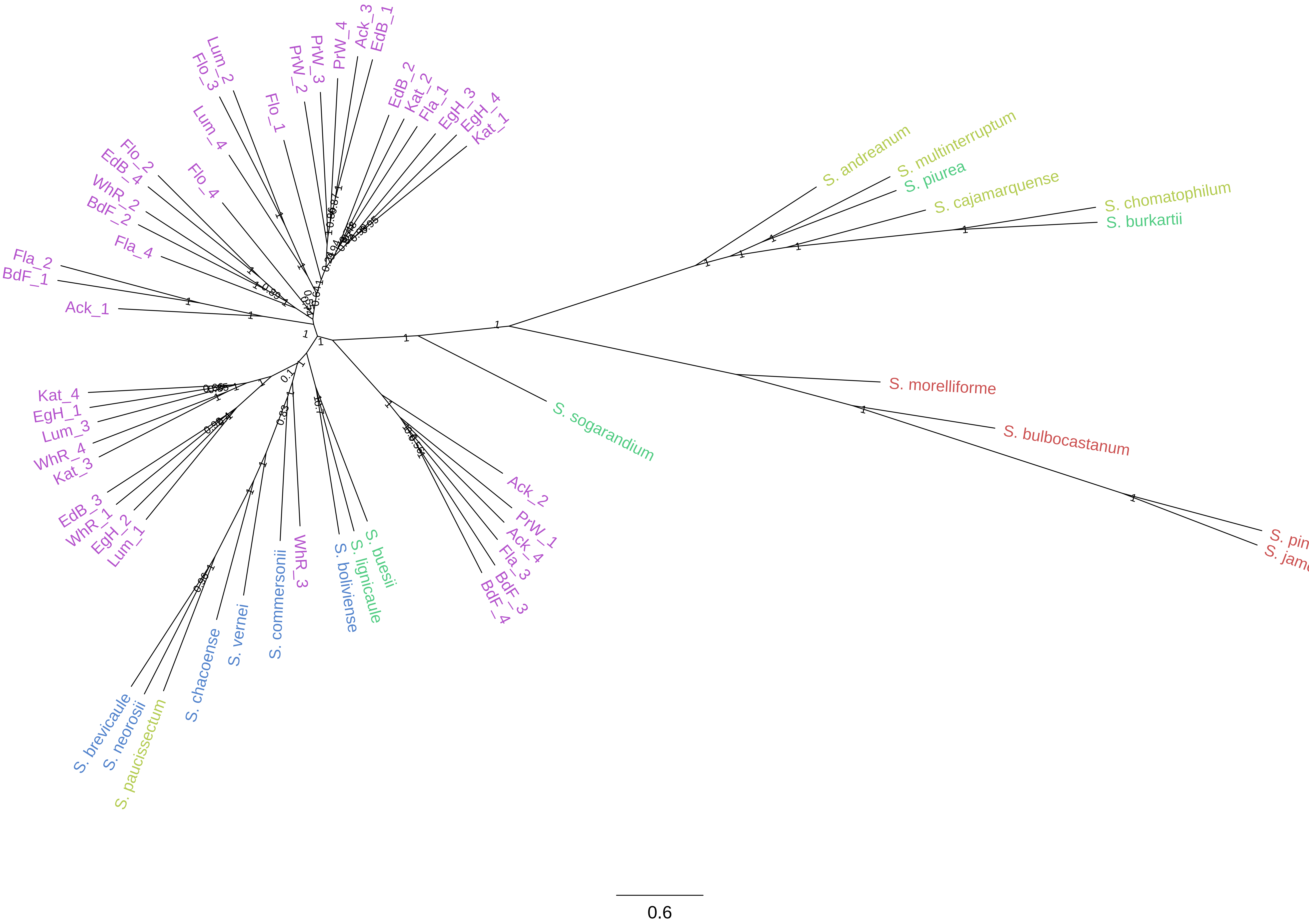

Supplement: Supplementary file 5 — Supplementary Figs. 3–14, 20, 21, 23–27 and 34–44. [file 41586_2025_8843_MOESM5_ESM.zip › suppl_figure_24/suppl_figure_24c.pdf]
